# Targeted Drug Delivery to the Spleen and Its Implications for the Prevention and Treatment of Cancer

**DOI:** 10.3390/pharmaceutics17050651

**Published:** 2025-05-15

**Authors:** Ikramy A. Khalil, Ahmed Faheem, Mohamed El-Tanani

**Affiliations:** 1 College of Pharmacy, Ras Al Khaimah Medical and Health Sciences University, Ras Al Khaimah P.O. Box 11172, United Arab Emirates; ahmed.faheem@sunderland.ac.uk (A.F.); eltanani@rakmhsu.ac.ae (M.E.-T.); 2 Faculty of Pharmacy, Assiut University, Assiut 71526, Egypt; 3 School of Pharmacy and Pharmaceutical Sciences, University of Sunderland, Sunderland SR13SD, UK

**Keywords:** spleen targeting, biodistribution, immune modulation, nanoparticles, cell-specific targeting, cancer immunotherapy

## Abstract

The spleen, the largest secondary lymphoid organ, plays several vital roles in the body, including blood filtration, hematopoiesis, and immune regulation. Despite its importance, the spleen has not received substantial attention as a target organ for drug delivery. Most systemically administered colloidal and particulate drug carriers are cleared from the blood by the liver and spleen, making these two organs potential targets for drug accumulation. While various systems have been developed to target the liver, there is an urgent need to design spleen-targeted drug delivery systems that can evade clearance and degradation while delivering drugs efficiently to their target cells in the spleen. Targeting the spleen holds great potential for the treatment of a range of diseases, including blood disorders, immune and inflammatory diseases, infectious diseases, and cancer. It is also crucial for the development of effective vaccines. In this review, we explore different approaches used to target the spleen after systemic administration, and we discuss the factors that shift the biodistribution of drug carriers from the liver to the spleen. We focus on cell-specific delivery within the spleen, strategies to avoid degradation, and methods to achieve the efficient intracellular delivery of various drugs and genes. We also highlight the therapeutic implications of spleen-targeted drug delivery systems, particularly for the prevention and treatment of cancer.

## 1. Introduction

The spleen is the largest secondary lymphoid organ in the body. It plays a vital role in blood filtration and the clearance of old and aging blood cells, blood storage and hematopoiesis, and immune regulation. It uniquely integrates innate and adaptive immunity through dynamic interactions among its different resident immune cells, including B and T lymphocytes, dendritic cells (DCs), and macrophages. Its unique structure of fenestrated blood vessels and specialized architecture enables the filtration of abnormal cells and any other foreign particles, such as pathogens or colloidal drug carriers [1,2,3]. Spleen disorders, including splenic malfunction, splenomegaly, or splenectomy, have wide effects on the body. The loss of splenic function (asplenia) causes impaired immune initiation and coordination against antigens [4,5]. The immunodeficiency and reduced antibody production result in a high risk of sepsis and high susceptibility to infections. It also causes the damaged or abnormal blood cells to persist and circulate longer, increasing the risk of blood clotting. Patients after splenectomy are at higher risk for infections. On the other hand, an overactive spleen (hypersplenism) can lead to the excessive destruction of blood cells, resulting in anemia, leukopenia, and thrombocytopenia. Splenomegaly, which is caused by liver diseases, infections, or cancers, exerts pressure on adjacent organs, causing pain and increased risk of splenic rupture [4,6].

Alongside the liver, the spleen plays an important role in clearing and degrading blood-borne antigens, including drug carriers [7]. Systemically administered drug carriers such as colloidal systems, liposomes, and nanoparticles (NPs) are rapidly cleared from the circulation through opsonization and phagocytosis by circulating phagocytes as well as those resident in the liver and spleen [8,9]. The continuous endothelium in most organs of the body makes it difficult for the drug carriers to get out of the blood vessels and reach target cells [10]. Having large fenestrations in their blood vessels, the liver and spleen tend to allow NPs and drug carriers to exit the bloodstream and accumulate within these organs [11]. It is, therefore, common to find most of the intravenously injected NPs eventually accumulating in the liver and spleen after a transient distribution to the lungs [12]. This fact of natural accumulation makes the liver and spleen attractive organs for potential efficient drug delivery and organ targeting. However, the main challenge is to avoid rapid degradation by liver and spleen macrophages to allow reaching other cells like hepatocytes in the liver and parenchymal and immune cells in the spleen, including DCs and B and T lymphocytes [13]. Here comes the importance of using cell-specific ligands and systems that can efficiently target specific cells in the liver or the spleen. For example, targeting spleen-resident DCs is crucial for developing vaccines against infections as well as cancers [14]. Recently, targeting B lymphocytes is receiving strong attention as a strategy of immune modulation [15,16,17]. Targeting T lymphocytes, however, remains a major challenge, particularly in delivering genes and nucleic acids and achieving efficient transfection in vivo [17].

While the liver has received the most attention as a target organ, with the development of various systems that can target liver cells, such attention is not given to the spleen as a potential target organ, despite its critical role in immune regulation and its natural ability to trap and filter colloidal drug carriers [2,18]. Compared to the liver, the spleen has larger fenestrations, and its circulation is open, which allows the filtration and trapping of relatively larger particles [3]. Therefore, higher attention should be given to the spleen as a relevant target organ in drug delivery, and a deep understanding of how to effectively deliver therapeutics to the spleen is needed to develop efficient spleen-targeted drug delivery systems. The most important challenge is to minimize the liver accumulation of drug carriers and shift their distribution from the liver to the spleen. The factors that affect drug carriers’ distribution, including particle size, surface charge, morphology, rigidity, lipid composition, and binding to protein corona, must be carefully considered.

The existing strategies for spleen targeting include passive and active targeting. Passive targeting is achieved by controlling the physical properties of the carriers, such as the particle size and surface charge [19,20]. Also, strong evidence exists regarding the effect of shape and rigidity of the systems and how they affect spleen delivery [20]. Polyethylene glycol (PEG) modification (PEGylation) is commonly used to decrease the clearance of the systems and extend the circulation time to enhance spleen uptake [21]. pH-responsive systems are developed to exploit the acidic environment in spleen macrophages to trigger drug activation and release [22]. Active targeting, on the other hand, can be achieved by using various ligands to target specific receptors on the surface of spleen cells [23]. These ligands include antibodies or antibody fragments, mannose, sialic acid analogs, aptamers, and short peptides. The lipid composition of lipid nanoparticles (LNPs) also has an important role in spleen uptake. Specific lipids have been shown to facilitate delivery to specific cell populations in the spleen [17], and proper optimization of the composition of LNPs is crucial for enhancing drug delivery, particularly in gene therapy and the development of mRNA vaccines [24,25].

In this review, we outline the anatomy and physiology of the spleen and emphasize its emerging role as a target organ for drug delivery in the treatment of various diseases. We explore the key factors that affect the distribution of drug nanocarriers in the body and how to promote spleen uptake over liver accumulation. We summarize the currently used spleen targeting strategies and approaches and discuss the challenges and future directions of spleen targeting. The therapeutic impact of spleen targeting is highlighted, especially its role in cancer prevention and treatment.

## 2. Spleen Anatomy

**Figure 1:**
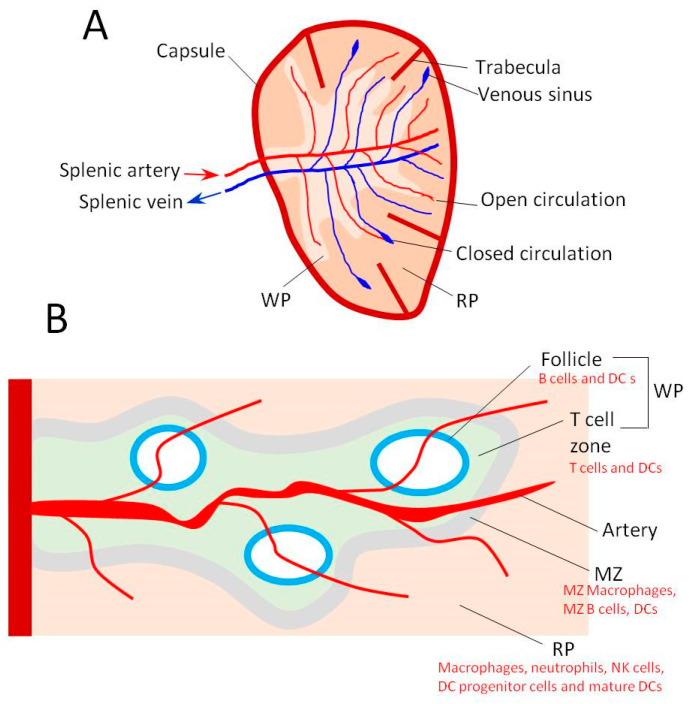
(**A**) Schematic representation of the structure of the spleen showing the capsule and trabeculae with the blood circulation. (**B**) Cross-section of the spleen showing the three main regions: white pulp (WP), marginal zone (MZ), and red pulp (RP). Types of cells in each region are shown.

The spleen is a large vascular organ, where blood vessels branch like a tree throughout the spleen parenchymal cells. It is enclosed by a connective tissue capsule, from which various trabeculae extend inward to the inside of the spleen to support the internal structure [26]. In the context of drug delivery, the spleen is typically divided into two main regions [5,27]; the red pulp (RP), which makes up approximately 75% of the spleen’s volume, and the white pulp (WP), comprising less than 25% of spleen volume, with a marginal zone (MZ) separating the two (Figure 1).

The RP contains a high density of RP macrophages, monocytes, and monocyte reservoir cells, along with other immune cells such as progenitor and mature DCs, neutrophils, and nature killer cells (NKCs) [4]. The WP consists of follicles rich in B cells and DCs, surrounded by a T cell zone populated by CD4+ and CD8+ T cells. The MZ harbors MZ macrophages, MZ B cells, DCs, and neutrophils [4]. During immune responses, MZ B cells migrate to the WP follicles for activation and antibody production, while DCs move to the T cell zone in the WP to initiate T cell activation [28]. T cell activation takes place after antigen presentation occurs in the T zone, while B cell activation and subsequent antibody production occur in the WP follicles.

Following the blood flow, the afferent splenic artery enters the spleen and branches within the WP, eventually ending as small capillaries in the RP [29]. Venous sinuses exist in the RP, where blood enters the venous network and returns to the circulation via the collecting vein [30]. Healthy blood cells and small drug carriers exit the arterial capillaries in the RB and squeeze into the venous sinuses to exit the spleen and re-enter the systemic circulation. In contrast, damaged and aging blood cells and larger or rigid drug carriers are unable to squeeze into the venous sinuses and remain trapped in the RB due to their rigid structure and large size [4]. The innate immune response is [13] primarily initiated in the RP, where most of the trapped cells and particles are phagocytosed by RP macrophages. Antigens that reach the MZ activate MZ macrophages, DCs, and B cells, which then migrate to the WP follicles and T cell zone to initiate the adaptive immune response. Drug delivery to the cells in the RP is relatively straightforward; however, this typically does not result in a strong immune response [31]. Effective immune-targeted drug delivery relies on delivering antigens to the MZ and the different regions of the WP, which is a relatively more challenging task. Achieving successful drug delivery for immune regulation requires that antigens escape from the blood capillaries in the MZ and WP and are selectively delivered to target cells of these areas, particularly DCs and B cells [3].

The architecture of the spleen is characterized by three key features that distinguish it from other organs. First, like the liver, the spleen contains fenestrated blood capillaries, allowing particles in the range of ~100–200 nm to exit the endothelium and reach cells in the MZ and WP follicles [31]. Second, the spleen has an open circulation in the RP, which enables larger particles to be trapped in the RP and interact with RP macrophages and other immune cells [32]. Third, the spleen exhibits slow blood flow, which permits high interactions between antigens and various resident spleen cells [33]. Effective vaccine development depends on interactions with antigen-presenting cells (APCs), including macrophages and DCs. Specific antigen delivery to B cells can initiate B cell activation and subsequent antibody production. However, direct T cell activation by antigens remains more challenging [17].

## 3. Spleen-Targeted Drug Delivery

Synthetic nanocarriers encapsulating various drugs, antigens, or nucleic acids are typically administered systemically. Larger particles (>1 µm) are initially trapped in the narrow capillaries of the lung following intravenous administration [34]. Systems carrying a net positive charge are more likely to accumulate in the lung due to nonspecific interactions with negatively charged serum proteins, which leads to aggregation and size enlargement, further promoting lung accumulation [34]. Eventually, these large particles or aggregates are degraded, and smaller particles are released into the systemic circulation, where they are rapidly cleared from the circulation by circulating macrophages and organs of the reticuloendothelial system (RES), such as the liver and spleen [35,36].

Most intravenously injected NPs are removed from the bloodstream within 15 min and accumulate (>80%) in the liver and spleen [12]. The liver’s high blood perfusion and fenestrated endothelium (~100–150 nm pores) facilitate the liver entrapment of nanocarriers [37]. Particles with a size smaller than 150 nm tend to preferentially be taken up by the liver, which represents a major barrier to spleen targeting. Smaller NPs of size < 60 nm show greater uptake in hepatocytes compared to larger particles (>150 nm) [38,39,40].

Particles larger than 200 nm are more likely to be trapped in the spleen due to its larger fenestrations (200–500 nm), combined with the slow blood flow in the spleen [41]. Therefore, an efficient spleen delivery of NPs requires efficient escape from uptake by circulating macrophages as well as liver uptake. The continuous endothelium and absence of fenestrations in organs other than the liver and spleen largely limit drug carrier access to those tissues [19]. Nevertheless, very small systems (<10 nm) are typically cleared by the kidney through glomerular filtration (GF) [42]. In the next section, we focus on the different strategies used to avoid uptake by circulating macrophages and the liver to achieve successful spleen targeting. We also highlight examples of nanocarriers that have successfully achieved spleen targeting.

### 3.1. Strategies for Spleen Delivery

#### 3.1.1. Manipulation of Physicochemical Properties

The manipulation of particle size, surface charge, particle rigidity, and protein corona binding can effectively enhance the spleen targeting of systemically administered NPs. As discussed earlier, particles with particle sizes slightly larger than 200 nm are preferentially trapped in the spleen. This is facilitated by the large fenestrations in spleen capillaries, the open circulation in the spleen, and its high and slow blood flow. In contrast, smaller particles (<150 nm) can pass through the endothelial barrier in the liver and reach liver hepatocytes [43].

Decuzzi et al. developed a predictive model to examine the effect of particle size on spleen delivery, suggesting that particles in the range of ~200–500 nm preferentially accumulate in the spleen [44]. Moghimi et al. demonstrated that particles > 150 nm ultimately accumulate in the spleen of rats 24 h after injection [45]. Klibanov et al. reported that PEG-coated liposomes larger than 200 nm tend to accumulate in the spleen rather than other organs [46]. Nakamura et al. observed that particles with a 450 nm diameter showed the highest spleen uptake [47]. Albanese et al. observed higher spleen uptake with particles in the ~100–200 nm range compared to particles with a size of ~50 nm [48]. Although these studies collectively confirm that particles > 200 nm are preferred for spleen delivery, it is important to note that surface charge, PEG modification, and lipid composition also play critical roles in influencing biodistribution and must be considered alongside particle size.

On the other hand, the surface charge also plays a critical role in the biodistribution of drug carriers and spleen-specific delivery [49]. Generally, positively charged particles tend to interact with serum proteins and become opsonized, leading to rapid clearance from the circulation by phagocytes. In contrast, slightly negatively charged systems have a higher likelihood of spleen retention, provided that other physicochemical properties are favorable for spleen delivery [49].

Kranz et al. demonstrated that the spleen targeting of LNPs can be achieved by adjusting the charge ratio between cationic lipid and RNA [50]. LNPs having a slightly negative charge extensively accumulated in the spleen compared to positive or highly negative NPs. Similarly, Kurosaki et al. found that modifying the surface charge of plasmid DNA (pDNA) complexed with polyethyleneimine (PEI) from positive to negative enhanced pDNA delivery to the spleen [51]. A study reported that negatively charged lipoplexes are delivered preferentially to the spleen, whereas positively charged particles are more likely to be delivered to the lung [52].

Cheng et al. introduced a class of molecules called SORT (selective organ targeting) to conventional LNPs [53]. The incorporation of negatively charged SORT molecules specifically enhanced spleen targeting. Likewise, the addition of negatively charged lipids to LNPs has been shown to improve the spleen delivery of mRNA-loaded NPs. However, the effect of charge is less consistent than that of particle size. Several studies have reported the opposite effect, where cationic systems are more delivered to the spleen. Several independent studies have shown that positively charged systems were more effectively delivered to the spleen [54,55,56]. Nevertheless, our own studies have demonstrated successful delivery of both pDNA and mRNA to the spleen using three different systems, all of which carried a slightly negative charge [15,16,17].

In addition to the size and surface charge, increased particle rigidity through lipid composition manipulation also contributes to spleen targeting [57]. Rigid NPs show greater retention in the spleen compared to more flexible LNPs [58]. The incorporation of cholesterol to increase the rigidity and stability of LNPs significantly affects the spleen delivery [17]. However, there appears to be an optimum cholesterol content that provides sufficient rigidity while still allowing for effective drug release. Controlling rigidity through cholesterol content improved spleen accumulation [59]. The rigidity tuning of LNPs enhanced both spleen targeting and T cell activation, where liposomes with higher rigidity were preferentially taken up by splenic macrophages [60].

Furthermore, binding to protein corona can also affect spleen targeting. The coat of protein corona that forms around NPs in the circulation can either enhance or suppress spleen delivery, depending on its composition. Walkey, C.D. et al. showed that protein corona formed around gold and silver NPs influenced organ distribution, with a strong correlation to spleen uptake [61]. T. Nissinen et al. developed a specific dual PEGylation (DPEG) method for mesoporous silicon (PSi) NPs [62]. The DPEG coating increased spleen uptake, which was mainly attributed to differences in protein corona composition between coated and uncoated particles. Y. Du et al. designed a protein corona-driven nanovaccine (PCNV) that was selectively taken up by APCs in the spleen [63]. The importance of protein corona in spleen targeting is further supported by studies from Yang et al. and Ritz et al. [64,65].

#### 3.1.2. Lipid Composition

**Figure 2:**
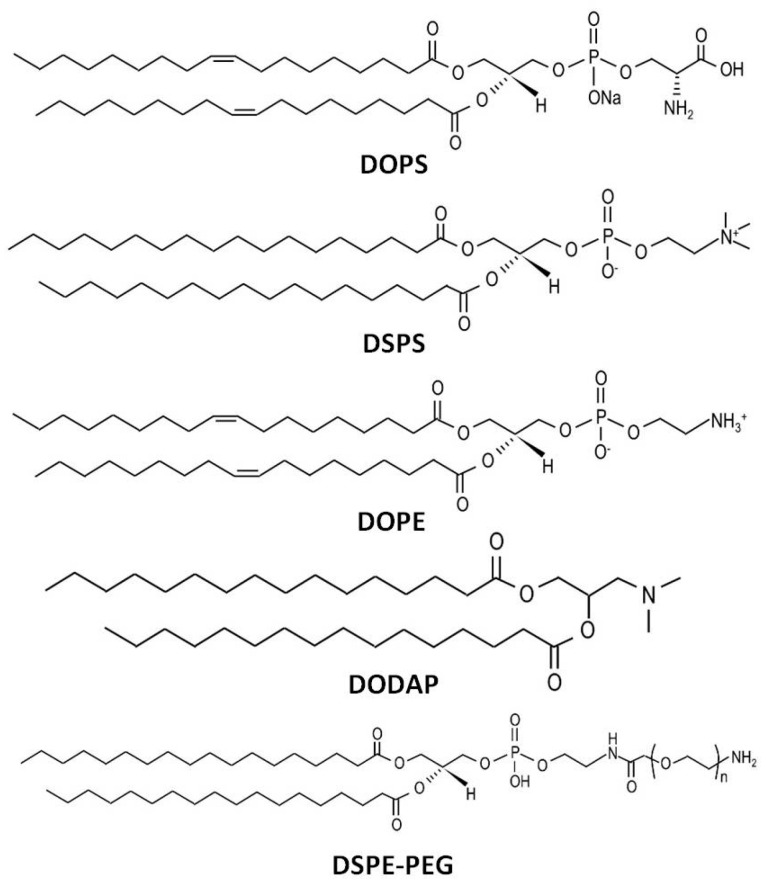
The structure of some lipids used for spleen-targeted delivery of LNPs.

The inclusion of specific lipids in nanoparticle formulations can promote spleen targeting (Figure 2). For example, incorporating phosphatidylserine into LNPs facilitates recognition by spleen macrophages and DCs [66]. pDNA-condensed NPs coated with 1,2-dioleoyl-sn-glycero-3-phospho-L-serine (DOPS) have demonstrated enhanced spleen delivery. Anionic lipids such as 1,2-dioleoyl-sn-glycero-3-phosphate (18PA) and 1,2-dimyristoyl-sn-glycero-3-phosphate (14PA) have been shown to facilitate spleen delivery and targeting [53]. Particles formulated with the helper lipid 1,2-dioleoyl-sn-glycero-3-phosphoethanolamine (DOPE) are more likely to be delivered to the liver, whereas those containing 1,2-distearoyl-sn-glycero-3-phosphocholine (DSPC) exhibit higher spleen delivery [67]. The interaction of NPs with serum apolipoprotein E (ApoE) appears to enhance liver uptake, as DOPE-containing NPs tend to bind more strongly to ApoE, facilitating their delivery to hepatic cells [68,69].

We have previously reported the development of pDNA nanocarriers with enhanced transfection in spleen cells, driven by the synergistic effect of an octa-arginine (R8) peptide and YSK05, a pH-responsive ionizable lipid [16,24]. The system was optimized for maximal spleen uptake and gene expression by modifying the lipid composition and employing a double-coating strategy to enhance spleen-selective transfection. The optimized system, which achieved the highest spleen delivery, had a particle size of ~202 nm and a negative surface charge. We also identified a novel lipid combination that can efficiently target spleen cells [17]. The main lipid was the ionizable lipid, 1,2-dioleoyl-3-dimethylammonium propane (DODAP), which surprisingly showed spleen-targeting properties only when combined with the helper lipid, DOPE. Maximum spleen transfection was observed when the DODAP/DOPE ratio ranged from 15/70 to 35/50. These optimized NPs had a diameter of ~270 nm and carried a slightly negative charge [17]. A similar DODAP/DOPE lipid composition was later used for mRNA delivery to spleen cells, resulting in the successful development of an mRNA-based cancer vaccine [15].

Other pH-sensitive ionizable lipids have also been used for the spleen targeting of nucleic acid carriers. While these lipids are primarily used to enhance endosomal escape [3], they also contribute to spleen delivery by modulating several physicochemical parameters. Fenton et al. successfully delivered mRNA to both liver and spleen cells using OF-Deg-Lin, an ionizable lipid that showed predominant gene expression in B cells of the spleen, despite effective delivery to liver cells [25]. It was suggested that the ester bonds in OF-Deg-Lin are degraded more rapidly in the liver than in the spleen. Similarly, S. Liu et al. used multi-tailed ionizable phospholipids (iPhos) in combination with DOPE to facilitate the spleen expression of mRNA [70]. The alkyl chain length of these lipids proved critical for spleen delivery, with lipids containing 13–16 carbon atoms showing the highest spleen activity [70]. Similarly, fatty acid-doped LNPs were shown to achieve spleen-selective mRNA translation [71]. Recently, Younis et al. reported a design of LNPs that target spleen APCs using CL15H6, a pH-sensitive ionizable lipid [72].

#### 3.1.3. Using Specific Ligands

Few ligands have been employed to target specific receptors expressed on various spleen cells, including DCs, macrophages, and B cells, thereby enhancing spleen accumulation and cellular targeting [73]. These ligands include antibodies, short peptides, and sugars. Macri et al. investigated the role of neonatal Fc receptor (FcRn) in targeting DEC205 and Clec9A receptors expressed on spleen DCs [74]. Mannose-modified nanocarriers are frequently used for targeting mannose receptors expressed in DCs and macrophages. Yuba et al. designed improved polysaccharide-based antigen carriers by introducing mannose residues to carboxylated curdlan [75]. mRNA lipoplexes were targeted to spleen DCs using a trimannose sugar tree, resulting in a robust T cell-mediated antitumor immunity [76]. Sialic acid (SA) and its derivatives can also be used for spleen targeting [77]. Earlier, the conjugation  of  MUC1  (tumor-associated  antigen)  with  anti-CD19  antibodies targeted B cells and generated anti-MUC1 antibodies and T cell-mediated immunity [78]. The use of synthetic polypeptides is another promising strategy for targeted drug delivery and immune regulation [79].

#### 3.1.4. PEG Modification

PEG modification is commonly used to reduce interactions with serum components, thereby increasing circulation time and enhancing the likelihood of organ delivery, provided that the NPs successfully escape from blood capillaries [21]. While PEG modification significantly improves tumor delivery in tumor-bearing mice, it also enhances spleen delivery. Alexis et al. demonstrated that intermediate PEG densities resulted in greater spleen accumulation [80]. PEG modification can facilitate delivery to both the liver and spleen, making careful optimization essential to minimize liver uptake and enhance the targeting of spleen cells. P. Laverman et al. showed that increasing the PEG molecular weight improved spleen accumulation, particularly through binding to WP macrophages [81]. Chiwoo Oh et al. found that liposomes modified with 5 kDa of PEG exhibited higher stability and greater spleen accumulation, compared to those modified with shorter PEG chains (2 kDa) [82]. Notably, a second dose of PEG-NPs tends to accumulate more in the spleen [83]. This is attributed to the generation of anti-PEG antibodies after the initial dose. These antibodies bind to subsequently administered PEG-NPs and the trigger rapid clearance of NPs, resulting in higher spleen accumulation compared to liver delivery.

#### 3.1.5. RBCs as Spleen Delivery Systems

Red blood cells (RBCs) can serve as carriers for the spleen-targeted delivery of drugs and diagnostic agents, primarily due to their natural spleen-mediated clearance, biodegradability, and long lifespan (~3 months) [84,85]. Aging or deformed RBCs are naturally filtered in the spleen and cleared by spleen macrophages, an attribute that is exploited for the spleen-specific uptake of drugs. However, the extent of RBCs aging and deformation influences organ distribution since normal RBCs tend to be delivered mainly to the lung tissue [86]. For spleen delivery, aged RBCs are frequently modified with other NPs, ligands or fused with lipid systems. Y. Zhai at al. fused RBCs with mannose liposomes and used them for cancer vaccination through targeting mannose receptors in APCs in the spleen [23]. L. Wang et al. prepared virus-like NPs attached to the surface of RBCs for spleen targeting, eliciting strong humoral and cellular antiviral immunity in mice [87]. X. Han et al. fused tumor cell membrane-associated antigens with RBCs, achieving spleen-targeted antigen delivery and tumor growth suppression [88]. A. Ukidve et al. developed RBC-Driven Immune Targeting (EDIT) to present bacterial pathogens to APCs in the spleen [89]. A summary of strategies used for spleen targeting is summarized in Table 1.

### 3.2. Cell-Specific Drug Delivery

We previously investigated which spleen cell populations are transfected by pDNA-loaded LNPs formulated with YSK05, a pH-sensitive ionizable lipid [16]. Using specific antibodies, we analyzed DNA delivery to various spleen cell types, including DCs, macrophages, B cells, and T cells. Our results showed that the majority of spleen macrophages (~80%) received pDNA, compared to ~50% in the case of B cells, with lower rates observed in DCs and T cells. However, given the higher abundance of B cells in the spleen relative to macrophages, the majority of the dose ultimately accumulated in B cells. Importantly, the delivery to B cells proved essential for immune activation, as blocking B cell uptake significantly reduced the immune response. Effective delivery to B cells also indicates that these LNPs are capable of escaping from blood capillaries in the WP. Similar findings were obtained with LNPs formulated using 1,2-dioleoyl-3-dimethylammonium-propane (DODAP), another ionizable lipid [17]. DCs, macrophages, and B cells, but not T cells, were transfected with pDNA. However, the quantification of gene expression in each cell type showed that most of the gene expression occurred in B cells.

Recently, targeting B cells in the spleen has received significant attention as a strategy for immune modulation [90]. Unlike T cells, B cells can be directly stimulated by antigens to produce antigen-specific antibodies. Moreover, B cells can act as APCs, similar to DCs and macrophages, and are capable of activating T cells to initiate cellular-mediated immunity. As discussed earlier, Fenton et al. used an ionizable lipid to deliver mRNA to B cells, resulting in both antibody-dependent and cellular immune responses [25]. Our findings showed that B cell activation produced an antitumor response, primarily through the activation of cytotoxic T lymphocytes, which confirms the role of B cells as APCs to activate cellular mediated immunity [17].

In the development of mRNA vaccines, DCs are often the primary target due to their status as professional APCs [91]. Lindsay et al. reported that spleen-resident DCs are transfected with messenger RNA (mRNA) following LNP administration [92]. Zhu et al. reported a screening method for the optimization of the helper lipid and lipid composition to enhance the delivery of mRNA to DCs in the spleen [93]. Bonifaz, L.C. et al. achieved robust humoral and cellular immunity through targeting DCs in the spleen with anti-DEC-205 antibody [94]. Ming et al. developed an RBC-driven DNA vaccine that targets DCs in the spleen [95]. In addition to DCs, macrophages, particularly those located in the RP or MZ, are also targeted as APCs. Oberli et al. showed that both DCs and macrophages in the spleen are transfected with mRNA [96]. Jain et al. prepared alginate-based NPs modified with tuftsin peptide to achieve active macrophage targeting [97]. In our previous work, we demonstrated that mRNA-loaded LNPs could transfect DCs, macrophages, and B cells in the spleen [15].

## 4. Therapeutic Implications of Spleen Targeting

The critical roles played by the spleen suggest that it can be targeted for the treatment of various spleen- and immune-related diseases. In this section, we focus on the therapeutic implications of spleen targeting in immune-related conditions. Given the spleen’s rich composition of immune cells, the targeted delivery of specific antigens to these cells is expected to stimulate the immune system, forming the basis for developing vaccines against infectious diseases as well as cancer. Conversely, suppressing the immune system through spleen targeting can also be employed for the treatment of autoimmune and inflammatory disorders.

Spleen-targeted delivery systems have shown promise in the development of cancer vaccines. Several protein and peptide antigens have been successfully delivered to spleen-resident immune cells to elicit antitumor immune responses [73,98]. Nanoparticles are widely used as antigen delivery systems as well as adjuvants in spleen-targeted systems [99]. T. Kurosaki et al. prepared a spleen-targeted, DNA-based cancer vaccine that achieved strong gene expression in the MZ of the spleen, leading to potent anti-tumor responses [51]. More recently, mRNA cancer vaccines have emerged as more efficient platforms, given that mRNA is translated in the cytosol and does not require nuclear entry. RNA–lipid polymer complexes (RNA-LPXs) have been utilized to express tumor antigens in spleen APCs, resulting in a robust antitumor effect [100]. In another study, RNA-LPXs modified with tri-mannose were developed to target mannose receptors on spleen DCs and macrophages, further enhancing targeted immune activation [76].

We have developed a successful cancer vaccine composed of DODAP/DOPE LNPs encapsulating OVA-encoding pDNA as a representative tumor antigen [17]. These LNPs were administered to mice prior to tumor cell inoculation, and tumor growth was subsequently monitored. Compared to several non-spleen-targeted systems, only the spleen-targeted LNPs elicited a robust prophylactic and therapeutic antitumor effect, highlighting the importance of developing spleen-targeted systems. In vivo imaging demonstrated strong spleen accumulation of these LNPs within 2 h of administration [17]. When analyzing the immune response, we found that spleen-targeted LNPs induced high OVA-specific antibody titers and potent OVA-specific cytotoxic T lymphocyte (CTL) responses. In contrast, other controls lacking spleen targeting capacity failed to generate significant immune responses. The expression of OVA antigens on the surface of APCs was confirmed using specific antibodies, indicating that these LNPs effectively transfected various APCs in the spleen and enabled the OVA antigen presentation to both B and T cells, ultimately leading to a strong immune response. Comparable results were observed with a similar spleen-targeted system encapsulating mRNA encoding the OVA tumor antigen [15].

Spleen-targeted drug delivery is particularly valuable for treating cancer types that involve immune system dysfunction, including hematological malignancies, such as different types of lymphoma, leukemia, and multiple myeloma. It is also beneficial in metastatic cancers with splenic involvement, such as melanoma, breast cancer, and lung cancer. Additionally, it enhances the effectiveness of immunotherapy-responsive cancers, such as cancers responsive to checkpoint inhibitors.

Spleen delivery can also be exploited to suppress immune responses through inducing systemic immune tolerance or selectively modulating immune activity by targeting APCs or regulatory T cells. Several spleen-targeted drug delivery systems have been developed for immune suppression as a method of treatment for autoimmune diseases. N. Pishesha et al. prepared RBCs functionalized with disease-associated autoantigens for inducing antigen-specific tolerance, demonstrating both prophylaxis and therapeutic efficacy in a type 1 diabetes mouse model [101]. C. Krienke et al. prepared an mRNA vaccine for the treatment of autoimmune encephalomyelitis [102]. J. Zhang et al. developed MicroRNA-125a-loaded polymeric NPs for the treatment of systemic lupus erythematosus (SLE), successfully delivering microRNA (miRNA) to splenic T cells in the SLE mouse model [103]. X. Chen et al. evaluated the therapeutic effects of a tolerogenic polypeptide vaccine composed of a multiepitope citrullinated peptide (Cit-ME) and rapamycin (Rapa) for the treatment of rheumatoid arthritis (RA), achieving therapeutic benefits [104]. U. Rauchhaus et al. demonstrated the spleen-targeted delivery of liposomes encapsulating dexamethasone, resulting in sustained therapeutic effects in a model of antigen-induced arthritis [105].

## 5. Discussion and Future Directions

Spleen-targeted drug delivery addresses a critical gap in how drug carriers are distributed after systemic administration. This topic is specifically important due to the unique vital role that the spleen plays in immune regulation, vaccine development, and disease management. Direct drug delivery to the spleen holds great promise for enhancing immunotherapy and improving cancer control. It also has significant potential applications in treating blood and infectious diseases.

Targeting drugs and antigens to spleen cells represents a promising strategy for developing both prophylactic and therapeutic vaccines against infections and cancer. Although there is currently no approved spleen-targeted vaccine, the mRNA vaccine based on RNA-LPXs is now in clinical trials and shows significant therapeutic potential [106]. Spleen targeting, however, is a challenging task, requiring several optimization steps to develop successful spleen delivery systems. Effective systems must avoid phagocytosis in circulation and liver uptake to reach the spleen. Further efforts are needed to understand and control the biodistribution of drug carriers in vivo. It is now clear that spleen uptake can be enhanced by controlling various physicochemical factors, such as particle size and surface charge. In addition, specific lipids and ligands can also play a crucial role in binding to spleen cells and facilitating spleen accumulation. Future research directions should focus on integrating these factors into a single system to achieve optimal spleen delivery. There is a need for using specific techniques for identifying new ligands that can bind to specific receptors on spleen cells.

For pDNA and mRNA vaccines, the nucleic acid load of the vaccines must be expressed or translated in target cells. This requires their internalization into cells, escape from endosomes, and eventual release from the carrier system [107]. Spleen accumulation alone does not guarantee successful antigen expression. In this context, B cell targeting is particularly important since the transfection of DCs and macrophages is generally more challenging [108]. Moreover, while DCs and macrophages account for approximately 10% of the total cells in the spleen, B cells are much more abundant [27]. To elicit a strong immune response, nanocarriers should ideally escape from blood capillaries in the follicles of the WP and the MZ to interact with DCs and B cells in these regions. Delivery to macrophages in the RB is less likely to trigger a robust immune response due to the extensive degradation of these carrier systems. Therefore, future work should focus on targeting specific cells in the spleen to avoid delivery to degrading macrophages and other cells that do not contribute significantly to immune modulation. The optimum use of PEG and studying factors affecting binding to protein corona and its correlation with spleen targeting will be crucial for developing more efficient systems. The proper selection of PEG molecular weight and density should control particle size and protein coronal binding towards enhanced spleen delivery. Moreover, using smart responsive systems could further improve delivery to specific cells within the spleen.

Limitations in spleen-targeted drug delivery impact the efficacy and safety of therapeutic systems (Table 2). The main challenge is the accumulation of systemically administered drug carriers in the liver, which reduces their availability in the spleen [39,40]. Another major limitation is the possibility of degradation in the spleen and failure to effectively delivering drugs or genes to specific cells in the spleen. Macrophages in the RP of the spleen effectively phagocytose drug carriers and degrade them. Additionally, NPs must escape from blood capillaries in the WP and MZ. Even when NPs are internalized into spleen cells, they must escape from endosomes to reach their intracellular target sites in the cytosol or the nucleus [109]. Furthermore, spleen delivery may trigger nonspecific inflammatory responses, which compromise drug safety. Understanding these limitations and finding innovative methods of overcoming them remains a true challenge in the field of spleen delivery to develop new effective and safe therapeutics. Developing new lipids and new ligands that can enhance spleen delivery and improve cellular uptake is a promising strategy for overcoming these challenges. Designing novel smart drug delivery systems that can be activated in the spleen is another promising approach. Optimizing the physicochemical properties of drug carriers and improving nanocarriers’ stability are also essential for effective delivery. Using new devices such as the microfluidic engineering of NPs is important for enabling the precise control of NP characteristics. Finally, innovative strategies to reduce unwanted immune responses, such as PEG coating and the use of cytokine inhibitors, are crucial for minimizing side effects.

The emerging use of pH-sensitive ionizable lipids for liver and spleen delivery is promising. These lipids are positively charged under acidic conditions and neutral under physiological pH. When injected, LNPs prepared from ionizable lipids are neutral or slightly negative. In the acidic environment of the endosomes, these lipids become positively charged, facilitating the endosomal escape of nucleic acids through fusion with endosomal membranes. This class of lipids offers a potential strategy for developing efficient nucleic acid vaccines in the future. However, avoiding liver uptake remains a challenging task.

## 6. Conclusions

Several formulation factors must be considered for developing successful spleen-targeted systems, including particle size, surface charge, particle rigidity, binding to protein corona, and using specific ligands for actively targeting receptors expressed on spleen cells. An ideal system should have a particle size of ~200–300 nm, a slight negative charge, bind selectively to specific protein coronas, and include components (lipids, polymers, or ligands) that target specific spleen cells. To expand the therapeutic applications of spleen-targeted systems, researchers must explore new techniques and strategies to not only enhance spleen accumulation but also to transfect specific cells in the spleen.

## Figures and Tables

**Table 1:** Strategies for spleen delivery.

Strategies	Examples	Description
1. Manipulation of physicochemical properties	Particle size	Particles > 200 nm are preferred for spleen delivery [46].
	Surface charge	Slightly negatively charged NPs are delivered preferentially to the spleen [51].
	Particle rigidity	Rigid NPs show greater retention in the spleen [58].
	Binding to protein corona	Binding to protein corona in the circulation can either enhance or suppress spleen delivery, depending on its composition [64].
2. Using specific lipids	DOPS	An anionic lipid that facilitates recognition by spleen macrophages and DCs [66].
	DSPC	A helper lipid that exhibits higher spleen delivery by inhibiting the interaction of NPs with serum apolipoprotein [67].
	DODAP	An ionizable lipid that showed spleen-targeting properties only when combined with the helper lipid DOPE [17].
	OF-Deg-Lin	An ionizable lipid that showed predominant gene expression in B cells of the spleen [25].
3. Using specific ligands	Mannose-modified nanocarriers are frequently used for targeting mannose receptors expressed in DCs and macrophages [75].	
4. PEG modification	Increasing the PEG molecular weight improved spleen accumulation, particularly through binding to WP macrophages [81].	
5. RBC-based delivery	RBCs can serve as carriers for spleen targeting due to their natural spleen-mediated clearance. RBCs are frequently modified with other NPs, ligands, or fused with lipid systems for enhancing spleen targeting [23].	

**Table 2:** Challenges in spleen-targeted drug delivery.

Challenge	Potential Solutions
1. Liver uptake before reaching the spleen	Optimize the particle size, surface charge, and rigidity of NPs. Use spleen-specific lipids and ligands to enhance spleen uptake. Develop lipids that are stable in the spleen but degrade in the liver. Design spleen-specific delivery systems. Improve cellular uptake in spleen immune cells. Apply PEG-coating to reduce liver uptake.
2. Clearance by spleen macrophages	Optimize surface charge. Apply PEG coating. Enhance cellular uptake in spleen immune cells.
3. Escape from blood capillaries	Optimize NP particle size and surface charge.
4. Targeting specific spleen cells	Develop novel lipids and ligands that target specific spleen cells. Design smart systems that are activated in specific spleen cells.
5. Intracellular degradation in lysosomes	Use novel pH-sensitive lipids with high endosomal escape ability. Incorporate fusogenic helper lipids. Apply pH-responsive polymers or peptides.
6. Unwanted immune response	Apply anti-inflammatory coatings. Use cytokine inhibitors and agents to modulate immune reactions. Develop controlled release formulations.
7. Low stability	Develop stable formulations with proper lipids and polymers. Optimize PEG coating. Use chemically modified nucleic acids for increased stability.
8. Scalability	Apply microfluidic technology for the precise and reproducible fabrication of NPs.

## Data Availability

All data and materials reported in this manuscript are available upon request from the corresponding authors.

## Abbreviations

The following abbreviations are used in this manuscript:

14PA	1,2-dimyristoyl-sn-glycero-3-phosphate
18PA	1,2-dioleoyl-sn-glycero-3-phosphate
APCs	Antigen-presenting cells
ApoE	Apolipoprotein E
Cit-ME	Multiepitope citrullinated peptide
CTL	Cytotoxic T lymphocyte
DCs	Dendritic cells
DODAP	1,2-dioleoyl-3-dimethylammonium propane
DOPE	1,2-dioleoyl-sn-glycero-3-phosphoethanolamine
DOPS	1,2-dioleoyl-sn-glycero-3-phospho-L-serine
DPEG	Dual PEGylation
DSPE-PEG	Distearoylphosphatidylethanolamine-polyethylene glycol
DSPC	1,2-distearoyl-sn-glycero-3-phosphocholine
EDIT	RBC-Driven Immune Targeting
GF	Glomerular filtration
iPhos	Multi-tailed ionizable phospholipids
LNPs	Lipid nanoparticles
LPXs	Lipid polymer complexes
mRNA	Messenger RNA
miRNA	MicroRNA
MZ	Marginal zone
NKCs	Nature killer cells
NPs	Nanoparticles
PCNV	Protein corona-driven nanovaccine
pDNA	Plasmid DNA
PEG	Polyethylene glycol
PEGylation	PEG-modification
PEI	Polyethyleneimine
PSi	Mesoporous silicon
R8	Octa-arginine peptide
RA	Rheumatoid arthritis
Rapa	Rapamycin
RBCs	Red blood cells
RES	Reticuloendothelial system
RP	Red pulp
SLE	Systemic lupus erythematosus
SORT	Selective organ targeting
WP	White pulp
